# GLP- 1R status using validated monoclonal antibody in 689 cases of neuroendocrine neoplasm and its correlation with somatostatin receptor scintigraphy, insulin production, and histological grades

**DOI:** 10.1007/s00428-025-04098-2

**Published:** 2025-04-26

**Authors:** Hirofumi Watanabe, Fumiyoshi Fujishima, Yuto Yamazaki, Masayuki Imamura, Susumu Hijioka, Kazuo Hara, Takamichi Kuwahara, Yasushi Yatabe, Kazuhiro Sakamoto, Hisashi Shiga, Tomohiro Kawaguchi, Hiroyoshi Suzuki, Yumi Kanbayashi, Akira Ohkoshi, Muneaki Shimada, Hiromichi Niikawa, Mami Sato, Atsushi Fujio, Toshihiko Masui, Yosuke Kasai, Hideki Ota, Hiroshi Ozawa, Hidenori Endo, Michiaki Unno, Hironobu Sasano, Takashi Suzuki

**Affiliations:** 1 Department of Pathology, Tohoku University Hospital, 1 - 1 Seiryo-Machi, Aoba-Ku, Sendai, Miyagi 980 - 8574 Japan; 2 Department of Surgery, Kansai Electric Power Hospital, Osaka, Japan; 3 Department of Hepatobiliary and Pancreatic Oncology, National Cancer Center, Tokyo, Japan; 4 Department of Gastroenterology, Aichi Cancer Center Hospital, Aichi, Japan; 5 Department of Pathology and Clinical Laboratories, National Cancer Center, Tokyo, Japan; 6 Department of Pathology, Osaki Citizen Hospital, Miyagi, Japan; 7 Division of Gastroenterology, Tohoku University Graduate School of Medicine, Miyagi, Japan; 8 Department of Stroke Neurology, Kohnan Hospital, Miyagi, Japan; 9 Department of Diagnostic Pathology, South Miyagi Medical Center, Miyagi, Japan; 10 Department of Dermatology, Tohoku University Graduate School of Medicine, Miyagi, Japan; 11 Department of Otorhinolaryngology, Head and Neck Surgery, Tohoku University Graduate School of Medicine, Miyagi, Japan; 12 Department of Gynecology, Tohoku University Graduate School of Medicine, Miyagi, Japan; 13 Department of Thoracic Surgery, Institute of Development, Aging and Cancer, Tohoku University, Miyagi, Japan; 14 Department of Breast and Endocrine Surgery, Tohoku University Hospital, Miyagi, Japan; 15 Department of Surgery, Graduate School of Medicine, Tohoku University, Miyagi, Japan; 16 Department of Surgery, Kyoto University Graduate School of Medicine, Kyoto, Japan; 17 Department of Diagnostic Radiology, Tohoku University Hospital, Miyagi, Japan; 18 Department of Orthopaedic Surgery, Tohoku Medical and Pharmaceutical University, Miyagi, Japan; 19 Department of Neurosurgery, Tohoku University Graduate School of Medicine, Miyagi, Japan

**Keywords:** Glucagon-like peptide 1 receptor, Immunohistochemistry, Neuroendocrine tumor, Insulin, Somatostatin receptor scintigraphy, Histological grades

## Abstract

Radiolabeled glucagon-like peptide 1 (GLP- 1) analog scintigraphy is a new, high-sensitivity imaging method for detecting small insulinomas. Somatostatin receptor scintigraphy (SRS) is an established method for detecting gastroenteropancreatic neuroendocrine tumors. However, small benign insulinomas are difficult to detect using SRS. Furthermore, GLP- 1 receptor (GLP- 1R) expression and SRS results may be inversely correlated. We identified 689 neuroendocrine neoplasms, including pancreatic neuroendocrine tumors (PanNETs) and neuroendocrine neoplasms originating from non-pancreatic sites, and performed GLP- 1R immunostaining. Among the non-insulinoma PanNETs, immunohistochemical insulin or proinsulin positive cases were categorized as Ins_pos_, and both negative cases as Ins_neg_. High prevalence of GLP- 1R expression was detected in PanNETs and duodenal NETs (34% and 53%, respectively). Some pulmonary NETs were GLP- 1R positive (9%). In contrast, neither GI-NEC excluding one case nor pulmonary NEC exhibited GLP- 1R expression. The percentage of GLP- 1R positive cases for Ins_pos_, Ins_neg_, and insulinoma was 31%, 0%, and 84%, respectively. Among PanNETs, GLP- 1R positive cases showed higher expression of insulin and proinsulin than negative cases. SRS-positive patients showed lower expression levels of insulin, proinsulin, and GLP- 1R than SRS-negative patients. The expression in PanNETs and duodenal NETs may be derived from the expression in their normal counterparts. Insulinoma and Ins_pos_ cases showed GLP- 1R expression. Furthermore, as GLP- 1R-positive patients showed significantly higher expression of insulin and proinsulin than GLP- 1R negative patients, GLP- 1R may also be associated with neoplastic insulin production and GLP- 1 analog scintigraphy may detect subclinical insulinomas. In addition, SRS-negative cases showed significantly higher GLP- 1R expression than SRS-positive cases. These results suggest the application potential of GLP- 1 analog scintigraphy in combination with SRS as a detection tool.

**Supplementary Information:**

The online version contains supplementary material available at 10.1007/s00428-025-04098-2.

## Introduction

Insulinomas are the most common functional pancreatic neuroendocrine tumors (PanNETs) that produce insulin and cause the hypoglycemic syndrome. Insulinomas are usually small in size, thereby complicating diagnosis using conventional imaging methods, such as CT, MRI, and EUS [1]. As even small insulinomas can cause clinical symptoms and be life-threatening if they cannot be surgically removed, imaging methods must be highly sensitive [2, 3]. Recently, radiolabeled glucagon-like peptide 1 (GLP- 1) analog scintigraphy has been introduced as a high-sensitivity imaging method for the detection of small insulinomas [3].

Somatostatin scintigraphy (SRS) is an established method for detecting gastroenteropancreatic neuroendocrine tumors. However, its detection is dependent on SSTR2 expression in the tumors, and small benign insulinomas are difficult to detect using SRS [1–4]. SRS detects biologically indolent with less sensitivity than GLP- 1 receptor (GLP- 1R) SPECT/CT [1], possibly because of lower SSTR2 expression [4, 5]. Furthermore, a previous study showed that in six malignant insulinomas, SRS-positive 3 cases were GLP- 1R scan-negative and three cases were GLP- 1R scan positive [3]. Therefore, GLP- 1R expression and SRS results were postulated to be inversely correlated; however, this hypothesis has not yet been confirmed. In addition, a recent study demonstrated the potential of GLP- 1R PET as a detection tool for pheochromocytomas, which are neuroendocrine tumors of the adrenal gland [6]. Therefore, neuroendocrine tumors of organs other than the pancreas might also express GLP- 1R; however, this has not been extensively studied.

GLP- 1R was associated with insulin secretion of β-cells of normal pancreatic tissues [7]. GLP- 1, an incretin hormone secreted from the enteroendocrine L cells in response to food intake, stimulates insulin release from β cells with connection to GLP- 1R [7]. Therefore, GLP- 1R expression might also be associated with insulin secretion in PanNETs, particularly insulinomas; however, this association has not been explored in detail. In addition, GLP- 1 has also been reported to augment cell proliferation and inhibit apoptosis in pancreatic endocrine β cells. These antiapoptotic actions are essential for the survival of β cells in response to cellular injury [8]. Other than β cells, GLP- 1 was also suggested to induce focal proliferation in the exocrine pancreas and, in the context of exocrine dysplasia, has been shown in previous studies to accelerate the formation of neoplastic PanIN lesions [9]. Moreover, the GLP- 1-based therapy for patients with diabetes potentially promotes pancreatic cancer [10]. In addition, GLP- 1R PET may be a potential tool for risk stratification of pheochromocytomas [6]. Therefore, the expression of GLP- 1R is postulated to be associated with neuroendocrine cell proliferation, progression, and the malignancy of neuroendocrine tumors; however, the relevant details are unknown.

The immunohistochemical expression of GLP- 1R has been studied using non-specific polyclonal antibodies [7]; however, in this study, we used an extensively validated monoclonal antibody (Mab3 F52) [7, 11]. Using this antibody, we explored the correlation between GLP- 1R expression and SRS and insulin production in PanNETs and compared its expression in PanNETs with that in neuroendocrine tumors of various organs. We also explored the association between its expression and histological grades.

## Materials & methods

### Samples and immunohistochemistry

We searched for information on NET, neuroendocrine carcinoma (NEC), carcinoid, middle ear adenoma/NET, olfactory neuroblastoma, paraganglioma/pheochromocytoma, cauda equina NET (CE-NET), medullary thyroid carcinoma, parathyroid adenoma, Merkel cell carcinoma, and pituitary adenoma/neuroendocrine tumors from pathology files at Tohoku University Hospital (Miyagi, Japan), Osaki Citizen Hospital (Miyagi, Japan), Aichi Prefectural Cancer Center Hospital (Nagoya, Japan), Noe Hospital (Osaka, Japan), Kansai Electric Power Hospital, and Kohnan Hospital (Miyagi, Japan). We excluded patients under 20 years of age at the time of surgery. Three pathologists (HW, FF, and HS) carefully reviewed the specimen histology and confirmed the diagnosis. In total, 689 cases were selected for immunohistochemical analysis, which included 108 PanNETs, 18 gastric NETs, 59 duodenal NETs, 1 NET of papilla Vater, 8 small intestinal NETs, 1 appendiceal NET, 79 rectal NETs, 1 NET of gallbladder, 2 NETs of extrahepatic bile duct, 27 gastrointestinal NECs, 23 pulmonary NETs, 5 mediastinum NETs, 22 pulmonary NECs, 1 renal NET, 9 urinary NECs, 3 NEC of uterine cervix, 3 middle ear NETs, 11 olfactory neuroblastomas, 1 NEC of pharynx, 90 paragangliomas/pheochromocytomas, 4 CE-NETs, 26 medullary thyroid carcinomas (MTC), 53 parathyroid adenomas, 6 parathyroid carcinomas, 20 Merkel cell carcinomas, and 108 pituitary neuroendocrine tumors. Eight cases (neuroendocrine carcinoma of the urine, uterine cervix, and pharynx and merkel cell carcinoma) were biopsied, and all other cases were resected. SRS was carried out in 17 patients with PanNETs. The results of the SRS were gained from radiological reports.

Serial sections of 10% formalin-fixed, paraffin-embedded (FFPE) tissues were prepared for subsequent analyses. A representative tissue section containing the tumor was selected for each case. Serial tissue sections with thickness within the range of 3–4 μm from the FFPE blocks were carefully prepared. The IHC protocols used in this study are summarized in Supplementary Table 1. We evaluated GLP- 1R immunoreactivity using a validated monoclonal antibody, 3 F52 [11–13]. The histological grades of pancreatic and gastrointestinal neuroendocrine tumors were determined by calculating the Ki- 67 labeling index (Ki- 67 Li) and the mitotic index according to WHO, as previously described [14–16].

The clinicopathological characteristics included in this study are summarized in Table 1. The research protocol of this study was approved by the institutional review boards of Tohoku University Graduate School of Medicine (2022–1–847, 2023–1–283) and the institutions mentioned above.

**Table 1:** Summary of clinicopathological findings of NENs in this study

Type of NEN	Total number (Male/Female)	Age Median (range)	Sampling method, n (%)	Histological grades	Other clinicopathological features, n (%)	GLP- 1R IRS, n									GLP- 1R IRS> 0
						0	1	2	3	4	6	8	9	12	
PanNET	108 (53/55)	60 (23–88)	Resection, n= 108 (100%)	G1, n= 63 (58%); G2, n = 43 (40%);G3, n = 2 (2%)	Hormonal activity: NF-PanNET n= 67 (62%); Insulinoma n = 31 (29%); PanNET, NOS n = 10 (9%)/Result of SRS: SRS positive, n = 11 (10%); SRS negative, n = 6 (6%); SRS NA, n = 91 (84%)/Hereditary background: MEN1, n = 7 (6%); VHL, n = 3 (3%); NA, n = 98 (91%)	71	3	4	7	8	2	9	0	4	37/108 (34%**)
Gastric NET	18 (15/4)	57 (23–88)	Resection, n= 18 (100%)	G1, n= 8 (44%); G2, n = 8 (44%); G3, n = 2 (12%)		15	0	1	1	0	1	0	0	0	3/18 (17%)
Duodenal NET	59 (36/23)	61 (31–81)	Resection, n= 59 (100%)	G1, n= 49 (83%); G2, n = 10 (17%)	Hereditary background: MEN1, n= 12 (20%); NF1, n = 1 (2%); NA, n = 46 (78%)	28	12	8	0	4	4	1	1	1	31/59 (53%)
NET of papilla Vater	1 (1/0)	61	Resection, n= 1 (100%)	G1, n= 1 (100%)		1	0	0	0	0	0	0	0	0	0/1 (0%)
SI-NET	8 (4/4)	62 (54–74)	Resection, n= 8 (100%)	G1, n= 5 (63%); G2, n = 3 (37%)		7	1	0	0	0	0	0	0	0	1/8 (13%)
Appendiceal NET	1 (0/1)	44	Resection, n= 1 (100%)	G1, n= 1 (100%)		1	0	0	0	0	0	0	0	0	0/1 (0%)
Rectal NET	79 (51/28)	60 (33–79)	Resection, n= 79 (100%)	G1, n= 59 (75%); G2, n = 19 (24%); G3, n = 1 (1%)		79	0	0	0	0	0	0	0	0	0/79 (0%)
NET of gallbladder	1(1/0)	58	Resection, n= 1 (100%)	G1, n= 62 (75%); G2, n = 20 (24%); G3, n = 1 (1%)		1	0	0	0	0	0	0	0	0	0/1 (0%)
NET of extrahepatic bile duct	2 (1/1)	54 (44–64)	Resection, n= 2 (100%)	G1, n= 1 (50%); G2, n = 1 (50%)		1	0	1	0	0	0	0	0	0	1/2 (50%)
GI-NEC	27 (20/7)	70 (43–86)	Resection, n = 27 (100%)		Site: Esophagus, n = 8 (30%); EG junction, n = 2 (8%); Stomach, n = 11 (41%); Papilla of vater, n = 1 (3%); Colon, n = 4 (15%); Rectum, n = 1 (3%)	27	0	0	0	1*	0	0	0	0	1/27 (4%)
Pulmonary NET	23 (11/12)	65 (36–81)	Resection, n = 23 (100%)	Typical carcinoid, n = 13 (57%); Atypical carcinoid, n = 7 (30%); Carcinoid, NOS, n = 3 (13%)		21	1	0	0	1	0	0	0	0	2/23 (9%)
Mediastinum NET	5 (4/1)	44 (36–57)	Resection, n = 5 (100%)	Atypical carcinoid, n = 5 (100%)		5	0	0	0	0	0	0	0	0	0/5 (0%)
Pulmonary NEC	22 (14/8)	71.5 (53–80)	Resection, n = 22 (100%)		Histology: Small cell carcinoma, n = 13 (59%); LCNEC, n = 9 (41%)	22	0	0	0	0	0	0	0	0	0/22 (0%)
Renal NET	1 (0/1)	46	Resection, n = 1 (100%)			1	0	0	0	0	0	0	0	0	0/1 (0%)
Urinary NEC	9 (8/1)	77 (67–87)	Resection, n = 6 (67%); Biopsy, n = 3 (33%)		Site: Ureter, n = 1 (11%); Urinary bladder, n = 5 (56%); Prostate, n = 3 (33%)	9	0	0	0	0	0	0	0	0	0/9 (0%)
NEC of uterine cervix	3 (0/3)	52 (35–62)	Resection, n = 2 (67%), Biopsy, n = 1 (23%),			3	0	0	0	0	0	0	0	0	0/3 (0%)
Middle ear NET	3 (2/1)	47 (46–52)	Resection, n = 3 (100%)			3	0	0	0	0	0	0	0	0	0/3 (0%)
Olfactory neuroblastoma	11 (8/3)	45 (31–75)	Resection n = 11 (100%)			11	0	0	0	0	0	0	0	0	0/11 (0%)
NEC of pharynx	1 (1/0)	58	Biopsy n = 1 (100%)			1	0	0	0	0	0	0	0	0	0/1 (0%)
Paraganglioma/Pheochromocytoma	90 (34/56)	51.5 (20–85)	Resection n = 90 (100%)		Histology: Paraganglioma, n = 28; Pheochromocytoma, n = 62	78	4	1	2	3	2	0	0	0	12/90 (13%)
Cauda equina NET	4(4/0)	51 (40–56)	Resection n = 4 (100%)			4	0	0	0	0	0	0	0	0	0/4 (0%)
Medullary thyroid carcinoma	26 (12/14)	57.5 (23–82)	Resection n = 26 (100%)			24	2	0	0	0	0	0	0	0	2/26 (8%)
Parathyroid adenoma	53 (16/37)	66 (25–86)	Resection n = 53 (100%)			52	0	0	0	0	1	0	0	0	1/53 (2%)
Parathyroid carcinoma	6 (3/3)	58.5 (57–75)	Resection n = 6 (100%)			6	0	0	0	0	0	0	0	0	0/6 (0%)
Merkel cell carcinoma	20 (7/13)	84 (56–93)	Resection, n = 17 (85%); Biopsy, n = 3 (15%)			20	0	0	0	0	0	0	0	0	0/20 (0%)
Pituitary NET	108 (53/55)	58 (21–79)	Resection n = 108 (100%)		PitNETof SF1 lineage/Gonadotroph PitNET, n = 55; PitNETof TPIT lineage/Corticotroph PitNET, n = 14; PitNETof PIT1 lineage/Lactotroph PitNET, n = 9; PitNETof PIT1 lineage/Somatotroph PitNET, n = 4; PitNETof PIT1 lineage/Mixed somatotroph-lactotroph PitNET, n = 1; PitNETof PIT1 lineage, NOS, n = 1; Mature plurihormonal PIT1-lineage PitNET, n = 3/Immature PIT1-lineage PitNET, n = 1; Null cell PitNET, n = 11; Plurihormonal PitNET, n = 2; Multiple synchromous PitNET, n = 3; PitNET, NOS, n = 4	108	0	0	0	0	0	0	0	0	0/108 (0%)

Abbreviation: *IRS* immunoreactivitiy score; *NEN* Neuroendocrine neoplasm; *NET* Neuroendocrine tumor; *NF* Non functional; *NOS* Not other specified; *SI* small intestinal; *GI* Gastrointestinal, *NEC* Neuroendocrine carcinoma; *LCNEC* Large cell neurondocrine carcinoma; *SRS* somatostatin receptor scintigraphy; *NA* Not available

^*^ Gastric NEC

^**^ Round off to the closest whole number

### Immunohistochemical staining for GLP1-R, insulin, and proinsulin immunoreactivity

**Fig. 1:**
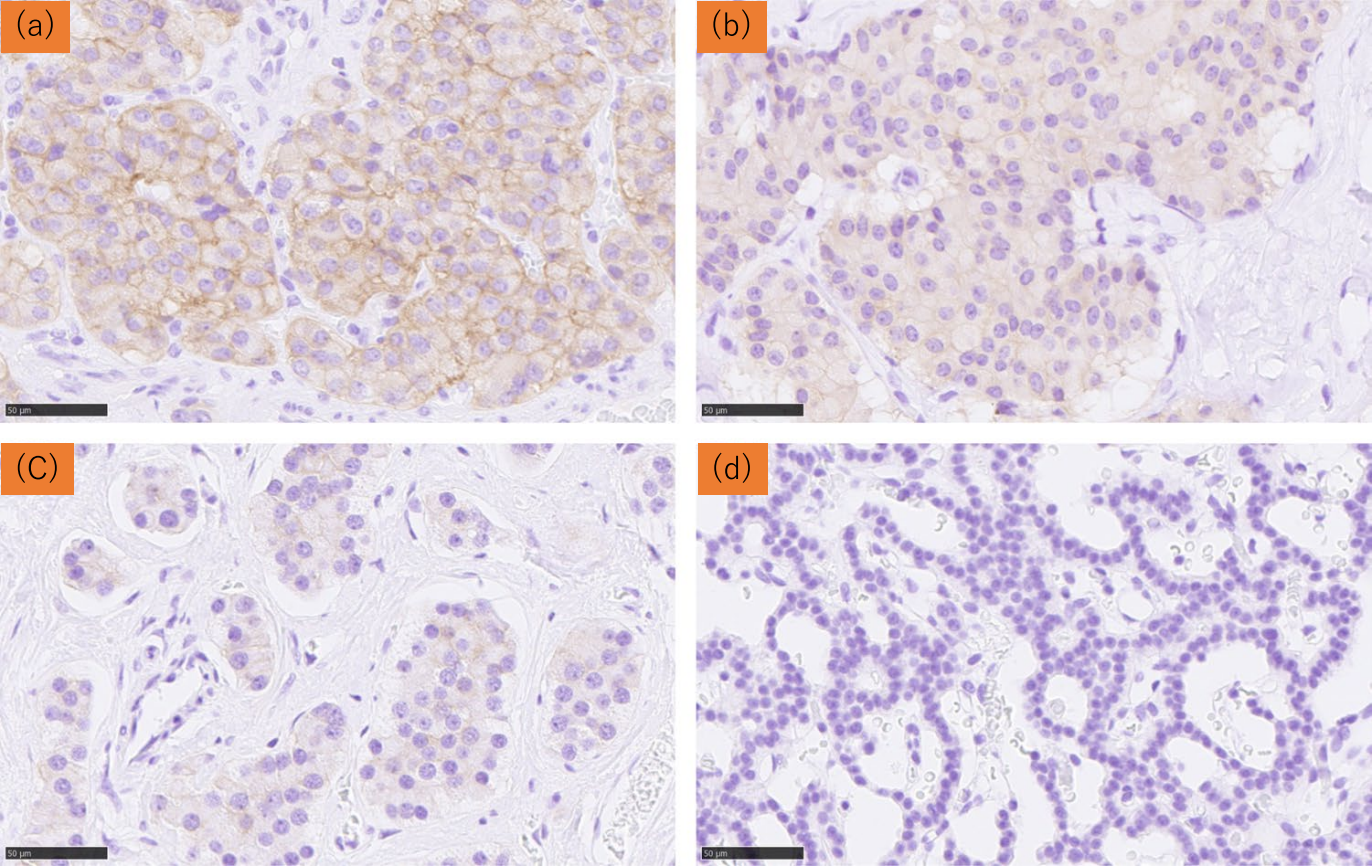
Representative illustrations of GLP- 1R immunohistochemistry. **a** Strong, (**b**) moderate, (**c**) weak (**d**) negative GLP- 1R immunoreactivity in the membrane of tumor cells

Membranous immunoreactivity of GLP- 1R and cytoplasmic immunoreactivity of insulin and proinsulin were evaluated semi-quantitatively using the immunoreactive score (IRS) [17]. IRS was calculated as reported in previous studies [17, 18]. An IRS ≥ 1 was defined as positive, and an IRS = 0 as negative [18, 19]. Representative images of GLP- 1R immunoreactivity are shown in Fig. 1.

### Statistical analyses

All statistical analyses were carried out in JMP Pro ver. 16.0.0 (SAS Institute, Cary, NC, USA). The percentage of GLP- 1R IRS positive cases were compared using the χ2 test. The differences of insulin-, proinsulin- and GLP- 1R-IRS were evaluated using the Mann–Whitney U test. The correlation between insulin-, proinsulin- and GLP- 1R-IRS were evaluated using Spearman’s test. Statistical significance was set at *P* < 0.05.

## Results

### GLP- 1R immunoreactivity

GLP- 1R expression in neuroendocrine neoplasms is summarized in Table 1. Briefly, the expression of GLP- 1R varied among different types of neuroendocrine neoplasms. High prevalence of GLP- 1R expression was detected in PanNETs and duodenal NETs (34% and 53%, respectively). Some of the gastric NETs, pulmonary NETs, paraganglioma/pheochromocytoma, and medullary thyroid carcinoma were GLP- 1R positive. Neither GI-NEC excluding one case nor pulmonary NEC exhibited GLP- 1R expression. All rectal NET and pituitary NET were negative for GLP- 1R expression.

Furthermore, we confirmed six cases with a hereditary background (Multiple Endocrine Neoplasia type 1, MEN1), showing NENs in multiple organ tissues. These results are summarized in Table 2. Of the six cases, three (Cases 1, 3, and 4) showed lesions in both tissues that were positive for GLP- 1R expression, one showed lesions in which one of the tissues was positive (Case 2), and two were negative (Cases 5 and 6).

**Table 2:** GLP- 1R expression in the cases with a hereditary background showing multiple NENs

Case No	Sex	Age	Hereditary background	Hormonal activity	Tissue	GLP- 1R IRS
1	F	52	MEN1	Gastrinoma	Duodenum	6
					Pancreas	12
2	M	46	MEN1	Gastrinoma	Duodenum	2
					Pancreas	0
3	M	39	MEN1	Gastrinoma	Duodenum	4
					Pancreas	8
4	M	31	MEN1	Gastrinoma	Duodenum	1
					Pancreas	8
5	M	67	MEN1	Gastrinoma	Duodenum	0
					Pancreas	0
6	M	52	MEN1	Gastrinoma	Duodenum	0
					Pancreas	0

Abbreviation: *IRS* immunoreactivitiy score; *MEN1* Multiple Endocrine Neoplasia type 1

### Correlation between GLP- 1R immunoreactivity and insulin and proinsulin immunoreactivity and SRS results in PanNETs

In PanNET cases other than insulinoma, insulin- or proinsulin-IRS-positive cases were categorized as Ins_pos_, both negative cases as Ins_neg_, referring to a previous study [18]. A comparison of GLP- 1R expression between Ins_pos_, Ins_neg_, and insulinomas is summarized in Table 3. The percentage of GLP- 1R positive cases for Ins_pos_, Ins_neg_, and insulinoma was 31%, 0%, and 84%, respectively. Expression of GLP- 1R was significantly different between these groups (Ins_pos_ vs. Ins_neg_, *p* < 0.0001; Ins_pos_ vs. Insulinoma, *p* < 0.0001; Ins_neg_ vs. Insulinoma, *p* < 0.0001).

**Table 3:** Association between insulin production and GLP- 1R expression

GLP1-R IRS > 0			*P*-value		
Non-insulinoma		Insulinoma			
Inspos	Insneg		Inspos vs.Insneg	Inspos vs.Insulinoma	Insneg vs.Insulinoma
11/35 (31%)	0/42 (0%)	26/31 (84%)	*P < 0.0001*	*P* < 0.0001	*P* < 0.0001

Abbreviation: IRS, immunoreactivitiy score

**Fig. 2:**
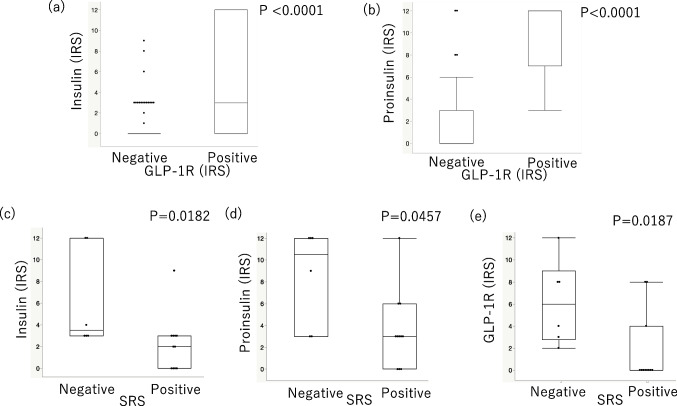
Box-plot graphs comparing immunoreactivity of GLP- 1R, insulin, proinsulin, and the result of SRS in PanNETs, GLP- 1R positive cases showed higher expression of (**a**) insulin (*p* < 0.0001) and (**b**) proinsulin (*p* < 0.0001) than GLP- 1R negative cases. SRS positive cases showed lower expression of (**c**) insulin (*p* = 0.0182), (**d**) proinsulin (*p* = 0.0457), and (**e**) GLP- 1R (*p* = 0.0187) than SRS negative cases

In PanNETs, GLP- 1R positive cases showed higher expression of insulin and proinsulin than GLP- 1R negative cases (Fig. 2a, b, *p* < 0.0001 and *p* < 0.0001, respectively). SRS-positive cases showed lower expression of insulin, proinsulin, and GLP- 1R than SRS-negative cases (Fig. 2c-e, *p* = 0.0182, *p* = 0.0457, and p = 0.0187, respectively). In addition, insulin- and proinsulin-IRS were both significantly correlated with GLP- 1R-IRS (Insulin-IRS: *p* < 0.0001, ρ = 0.6011; Proinsulin-IRS: *p* < 0.0001, ρ = 0.7348) analyzed by Spearman’s test.

### Correlation between GLP- 1R expression and the histological grade

We explored the correlation between GLP- 1R immunoreactivity and histological grade in PanNETs and duodenal NETs. The results are summarized in Supplementary Table 2. No statistically significant differences were detected in the histological grade in all cases.

## Discussion

In this study, we first evaluated GLP- 1R immunoreactivity using a validated monoclonal antibody, 3 F52 [11], in a large number of neuroendocrine tumors of the whole body. Furthermore, we explored the association between the primary sites, insulin or proinsulin productivity, SRS results, and histological grades.

In epithelial NENs, PanNETs and duodenal NETs show relatively high prevalence of GLP- 1R expression (34% and 53%, respectively, Table 1). In non-epithelial NENs, Paraganglioma/Pheochromocytoma also showed GLP- 1R expression (13%, Table 1). Previous studies showed the expression of GLP- 1R in insulinomas and paraganglioma/pheochromocytomas, but not in pituitary adenomas and pulmonary small cell carcinomas [20, 21] and the results of these previous studies are comparable with those of this study. Previous studies have observed that GLP- 1R expression was detected by autoradiography in normal tissues such as islets of the pancreas and Brunner’s glands of the duodenum [20, 22]. PanNETs and duodenal NETs often arise from neuroendocrine cells in the islets and Brunner’s glands, respectively. Therefore, we hypothesized that the expression in these tumors results from the expression native to their normal counterparts. GLP- 1R expression was also detected in 2/26 cases (8%; Table 1) of MTCs. Previous reports have demonstrated that 2/10 (20%) sporadic MTC cases and 0/10 (0%) MEN2 MTCs exhibit GLP- 1R expression [20, 23], which is consistent with our results. In addition, a previous study showed that C cells in human thyroid tissue do not express GLP- 1R [23]. MTCs showed hardly any GLP- 1R expression, possibly because MTCs might inherit C cells that do not show GLP- 1R expression. A larger sample of such cases is required for further clarification.

GLP- 1R expression was compared with insulin or proinsulin production and the results of SRS. Both insulinoma and Ins_pos_ cases exhibited GLP- 1R expression (Table 3). Furthermore, GLP- 1R-positive cases showed statistically significantly higher expression of insulin and proinsulin than GLP- 1R negative cases, so GLP- 1R could be associated with insulin production in PanNETs like β cells in pancreatic islets [8]. In addition, SRS-negative cases showed significantly higher expression of insulin, proinsulin, and GLP- 1R than SRS-positive cases, which is consistent with the results of a previous study that showed an inverse correlation between the results of the GLP- 1 receptor and somatostatin receptor subtype 2 scan [3]. Therefore, glucagon-like peptide 1 (GLP- 1) analog scintigraphy is likely not only a sensitive imaging method for the detection of small insulinomas [3], but also for subclinical insulinomas, and SRS-negative PanNETs. Furthermore, in certain patients with a hereditary background, MEN1 and multiple NET lesions showed GLP- 1R expression (Table 2). In addition, a previous report has suggested a detection tool targeting increased GLP- 1R expression in early lesions of MEN1 pancreas [24]. The usefulness of GLP- 1 analog scintigraphy as a tool for detecting multiple NETs should also be explored.

To investigate the relationship between GLP- 1R expression and malignancy, we examined the association between GLP- 1R and histological grade. Specifically, we studied PanNETs and duodenal NETs, which have relatively high GLP- 1R expression rates, but found no correlation between GLP- 1R and histological grade. Previous studies on PanNETs, specifically insulinomas, have demonstrated a relationship between GLP- 1R expression and prognosis, indicating that the lack of GLP- 1R expression is associated with poor prognosis [25]. The association between GLP- 1R expression and malignancy has been unclear. So further investigation is needed to clarify the relationship between GLP- 1R expression and malignancy.

The limitations of the present study are that GLP- 1R, insulin, and proinsulin were evaluated only immunohistochemically. We used samples collected between 2000 and 2024, so the results of immunohistochemistry might be affected by the aging of the tissue blocks. In addition, the cases were not collected consecutively and the number of cases tested via SRS was small, leading to the risk of selection bias. We might include patients with a history of endocrine disorders, which could be associated with insulin or GLP- 1R expression.

In conclusion, we suggested that GLP- 1R is expressed not only in insulinomas but also in subclinical insulinomas in PanNETs. In addition to PanNETs, duodenal NETs show high prevalence of GLP- 1R expression. Furthermore, SRS-negative PanNET cases show higher GLP- 1R expression; therefore, we propose the potential use of GLP- 1 analog scintigraphy as a detection tool in combination with SRS; however, this result warrants further analyses, including radiological studies.

## Supplementary Information

Below is the link to the electronic supplementary material.

Supplementary file1 (XLSX 10 KB)

Supplementary file2 (XLSX 10 KB)

## Data Availability

The data used in this study are available from the corresponding author. The data are not publicly available due to ethical restrictions.
